# Body weight dissatisfaction and communication with parents among adolescents in 24 countries: international cross-sectional survey

**DOI:** 10.1186/1471-2458-9-52

**Published:** 2009-02-06

**Authors:** Haleama Al Sabbah, Carine A Vereecken, Frank J Elgar, Tonja Nansel, Katrin Aasvee, Ziad Abdeen, Kristiina Ojala, Namanjeet Ahluwalia, Lea Maes

**Affiliations:** 1 Department of Public Health, Faculty of Medicine, Ghent University, Ghent, Belgium; 2 Nutrition and Health Research Institute, Al Quds University, Al Quds, Palestine; 3 Department of Psychology, Carleton University, Ottawa, Canada; 4 National Institute of Child Health and Human Development, NIH, DHHS, Bethesda, MD, USA; 5 INSERM U558, Faculty of Medicine, Toulouse, France; 6 Department of Chronic Diseases, National Institute for Health Development, Tallinn, Estonia; 7 Department of Health Sciences, Research Center for Health Promotion, University of Jyväskylä, Jyväskylä, Finland

## Abstract

**Background:**

Parents have significant influence on behaviors and perceptions surrounding eating, body image and weight in adolescents. The aim of this study was to examine the prevalence of body weight dissatisfaction, difficulty in communication with the parents and the relationship between communication with parents and adolescents' dissatisfaction with their body weight (dieting or perceived need to diet).

**Methods:**

Survey data were collected from adolescents in 24 countries and regions in Europe, Canada, and the USA who participated in the cross-sectional 2001/2002 Health Behaviour of School-Aged Children (HBSC) study. The association between communication with parents and body weight dissatisfaction was examined using binary logistic regression analysis.

**Results:**

Body weight dissatisfaction was highly prevalent and more common among girls than boys, among overweight than non-overweight, and among older adolescents than younger adolescents. Difficulty in talking to father was more common than difficulty in talking to mother in all countries and it was greater among girls than among boys and increased with age. Difficulties in talking to father were associated with weight dissatisfaction among both boys and girls in most countries. Difficulties in talking to mother were rarely associated with body weight dissatisfaction among boys while among girls this association was found in most countries.

**Conclusion:**

The findings suggest that enhanced parent communication might contribute in most countries to less body dissatisfaction in girls and better communication with the father can help avoiding body weight dissatisfaction in boys. Professionals working with adolescents and their families should help adolescents to have a healthy weight and positive body image and promote effective parent – adolescent communication.

## Background

Body weight dissatisfaction and fear of fatness in early adolescence are important risk factors for disordered eating [1,2] and are considered to be significant health concerns among health professionals worldwide [3]. Being thin is desired within Western societies, and many normal weight adolescents, especially girls, perceive themselves as overweight [1,4] and try to lose weight to achieve the socially endorsed ideal of a beautiful body [5]. Studies have shown variability in body size preference and in body image dissatisfaction among children and adolescents based on ethnicity, body mass index, gender, age, pubertal status, and family connectedness [2,6,7]. A positive association has been found between body mass index (BMI) and body image dissatisfaction among youth [8]. For both genders, the desire to change shape or weight is common [9]; however, gender differences in body dissatisfaction have been noted in early adolescence with girls being more dissatisfied with their bodies than boys [1,4,7,10,11].

In addition, research on body dissatisfaction indicates that adolescents are often split between those who desire to lose weight versus those who desire to gain weight [9], therefore body weight dissatisfaction is associated with perceived under- or overweight. The definition of body dissatisfaction varies across studies; some studies focused on body image or body shape dissatisfaction (e.g., how adolescents consider the appearance of their bodies) while other studies have focused on the perception of body weight (e.g., how adolescents feel about their weight and if they feel that they are overweight or not). In one study, body dissatisfaction was defined as the affective component of the multidimensional construct of body image, that is how individuals feel about their body [12]. In the present study we considered body weight dissatisfaction from an overweight/obesity perspective, i.e. adolescents reporting to be on a diet to lose weight or need to lose weight were considered to be dissatisfied with their weight.

The prime context where the child lives and develops is the family. It is in this context that the cognitive, social and emotional development starts [13]. The family is the cornerstone for promoting healthy behaviors and is an important source of social support. Parents are the main source of children's health care information [14]; therefore parents can play an important role in reinforcing positive influences and filtering out negative influences on their children [12]. In particular, mothers are the primary source of health-related information [14] and have a strong influence on adolescent females' attitudes and behaviors [15]. Poor parent-adolescent relationships could impair adolescents' psychological adjustment and increase their risk of psychopathology [16]. Adolescents with more supportive parents are less often depressed or psychologically distressed [16]. In addition, qualitative research among adolescents has shown that a negative relationship with parents is related to weight dissatisfaction [17].

An indicator for the quality of the parent-child relations and of parental involvement is the communication between the adolescent and the family members. Communication with family members has been shown to work well as a general measure of family relations and a good measure of the quality of the parent-child-relationships, showing high correlations with several measures of attachment to parents [18].

The study aimed at assessing the prevalence in boys and girls of self reported body weight dissatisfaction and the prevalence of difficulties in talking to father and mother, in 24 countries/regions in Europe, Canada and USA participating in the HBSC 2001/2002 survey. Because parents have been found to have a significant influence on behaviors [15,19] and perceptions surrounding eating [20-22], body image [11], and weight [17,23] in adolescents, the aim of this study was to investigate cross culturally the relationship between adolescents self-reported body weight dissatisfaction and mother/father-adolescent communication. We hypothesized that both girls and boys who indicated difficulty in talking to their father or mother would be more likely to report body weight dissatisfaction.

## Methods

Data were obtained from the Health Behaviour in School-aged Children (HBSC) study 2001/2002, a cross-sectional survey that was carried out in 35 countries and regions across Europe, Canada, and USA, with the collaboration of the World Health Organization. The 2001/2002 survey included rich countries (such as Canada, Norway, Sweden and the United States), poor countries (such as Lithuania, the Russian Federation and Ukraine) and middle-income countries (such as Austria, Belgium- both the Flemish- and French-speaking populations, Croatia, the Czech Republic, Denmark, England, Estonia, Finland, France, Germany, Greece, Greenland, Hungary, Ireland, Israel, Italy, Latvia, Malta, the Netherlands, Poland, Portugal, Scotland, Slovenia, Spain, Switzerland, Macedonia, and Wales). Children were selected using a clustered sampling design, where the sampling unit was either the school class or the school. More than 160,000 young people took part and approximately 1500 respondents in each of the three age groups were targeted in every country.

This cross-sectional survey of 11-, 13- and 15-year-old schoolchildren is undertaken every four years. The goal of the HBSC study is to identify youth health indicators and the factors that influence them. School-based questionnaires were administered in classroom settings using standardised instructions. All the countries carried out the data collection in accordance with the international study protocol, providing a strong basis for international comparisons [24]. More details about data collection methods can be found on the HBSC website at . Countries/regions with more than 20% of missing values for BMI were excluded, resulting in a final sample of children from 24 countries/regions.

### Measures

#### Body Weight Dissatisfaction (desire to lose weight)

Categories were derived from the following question: *At present are you on a diet or doing something else to lose weight? *Respondents selecting 'no, my weight is fine' were classified as satisfied with weight; respondents selecting either 'no, but I need to lose weight' or 'yes,' were classified as dissatisfied with weight. Those who reported needing to put on weight (9.4%) were excluded from this analysis, because the main focus of this analysis was to examine the relationship of communication with parents and weight dissatisfaction (dieting or perceived a need to diet) from an overweight/obesity perspective. The percentage of missing data for this measure was 0.7% randomly distributed over countries and ranged from 0.1–1.7%.

#### Communication with Parents (talking to mother/father)

Communication with mother and father was assessed separately with two items, worded "*How easy is it for you to talk to your mother/father about things that really bother you?" *Response options were: "very easy," "easy," "difficult," "very difficult," and "don't have or see this person." Responses of "very easy" and "easy" were categorized as easy to talk; responses of "difficult," "very difficult," or "don't have or see this person" were categorized as difficult to talk.

We assumed that if adolescents do not have or see their father/mother then they are not supported. In addition the percentages of dissatisfied with their body image, were quite similar for those who responded not to have/see a father/mother and those who responded to have difficulty in talking to father/mother (respectively for father: 42.4% and 45.3% and for mother: 40.3% and 45.5%), while much lower percentages were found in those who responded that it was easy to talk to their parents (32.1% for talking to father and 35.5% for talking to mother). The percentages of missing data for both talking with father and talking with mother were 2.8%, ranging from 0.1–5.5% over the countries.

#### Body Mass Index (BMI)

BMI was calculated using self-reported weight and height (kg/m^2^). Adolescents' weight status was categorized by means of age- and gender-specific BMI international cut-off points recommended for use in international comparisons [25]. In the present study, the group of overweight adolescents includes obese. Adolescents who did not report their weight or/and height were excluded from the analysis because BMI could not be calculated. Missing values of BMI for the included countries/region ranged from 2.9% – 18.4%.

### Statistical analysis

Data analysis was performed using the SPSS Version 12. Binary logistic regression analyses were used to investigate the associations between communication with father/mother and body weight dissatisfaction, controlling for communication with the other parent, age, and BMI. Analyses were conducted separately for boys and girls. Probable interaction effects between age and weight dissatisfaction on communication with parents controlling for BMI were examined separately for boys and girls. In addition, interaction effects were examined between overweight status and weight dissatisfaction. A significance level of 0.05 was used for all statistical analyses and odds ratios were considered significantly different from 0 if 95% confidence intervals did not include 1.0.

## Results

In most countries, no interaction effect was found between age and weight dissatisfaction controlling for BMI. Exceptions among girls for communication with father were observed in Canada (*p *= 0.015), Switzerland (*p *< 0.001), and Finland (*p *= 0.004), and among boys only in Portugal (*p *= 0.018). For communication with mother, this interaction was observed only in Slovenia (*p *= 0.037) and Ukraine (*p *= 0.047) among girls. There was no interaction between overweight status and weight dissatisfaction across gender and countries.

### Prevalence of body weight dissatisfaction by demographic characteristics (country/region, gender, age and weight status)

Body weight dissatisfaction was found in many adolescents, although this also varied by country, gender, age, and weight status. Girls were more likely to report body weight dissatisfaction than boys. Adolescents' age and overweight status were positively associated with body weight dissatisfaction in almost all countries (Table 1). Among adolescent boys the highest rates of body weight dissatisfaction were found in Italy (39.9%), USA (37.7%), and Greece (35.2%), while among girls the highest rates of body weight dissatisfaction were found in Czech Republic (61.8%), Slovenia (56.8%), and Italy (55.2%) (Table 1).

**Table 1:** Body weight dissatisfaction by gender, age and weight status

**Country**	**Body weight dissatisfaction**							
	
	**N**	**Boys** **%**	**Girls** **%**	**11 y** **%**	**13 y** **%**	**15 y** **%**	**Non-Overweight** **%**	**Overweight** **%**
Belgium-Flanders	5825	23.8	40.0***	29.5	32.6*	35.2***	26.4	80.5***
Canada	4017	27.3	43.1***	28.8	37.5***	44.4***	26.6	67.2***
Switzerland	4252	23.1	42.7***	28.3	35.0***	35.7***	28.0	79.3***
Croatia	3669	34.0	51.3***	39.9	40.6	48.4***	37.1	76.7***
Czech Republic	4378	34.1	61.8***	43.2	52.0***	51.9***	45.1	81.9***
Germany	5170	30.7	46.0***	33.6	40.2***	42.5***	31.4	78.6***
Denmark	4217	28.3	45.4***	32.4	38.2**	41.0***	29.2	85.4***
Estonia	3723	19.0	41.9***	23.2	32.7***	36.4***	27.9	68.4***
Finland	5020	22.7	40.8***	26.8	34.5***	34.2***	24.0	73.9***
France	7321	28.7	50.5***	34.4	41.9***	43.2***	33.8	84.2***
Greece	3368	35.2	51.9***	41.8	44.0	45.8*	35.4	78.9***
Italy	3833	39.9	55.2***	41.1	48.7***	56.1***	39.2	84.7***
Latvia	3108	15.5	40.1***	22.0	29.9***	34.5***	26.7	62.7***
Macedonia	3471	28.8	48.0***	33.0	39.6**	44.3***	34.1	72.1***
Netherlands	3885	20.7	34.1***	22.8	30.1***	29.7***	22.5	70.9***
Norway	4458	23.0	46.1***	25.9	36.7***	41.3***	29.5	64.8***
Poland	5569	27.8	52.1***	33.4	41.6***	46.3***	36.2	77.6***
Portugal	2497	23.9	44.0***	30.1	36.5**	38.6***	26.4	63.1***
Russia	7383	15.4	36.0***	23.8	26.3*	30.3***	24.5	56.6***
Sweden	3446	20.9	38.2***	21.5	33.8***	36.0***	24.1	63.6***
Slovenia	3634	30.1	56.8***	37.9	46.2***	47.9***	36.3	81.6***
Ukraine	3589	14.1	43.8***	23.9	28.8**	36.5***	29.0	54.0***
USA	4563	37.7	51.0***	38.9	41.8	54.9***	33.0	73.8***
Wales	3586	33.4	53.7***	38.8	45.0**	47.3***	33.1	78.4***

References: Age = 11y, Gender = boys, Weight status = non-overweight

Overweight and non-overweight were calculated using international cutoffs for BMI. BMI was calculated based on self reported weight and height

* *p *< 0.05, ** *p *< 0.01, *** *p *< 0.001

### Prevalence of difficulty to communicate with parents by demographic characteristics (country, gender, age and weight status)

In general, difficulty in talking to father was more common than difficulty in talking to mother. Difficulty in talking to either parent varied by country, gender and age; difficulty in talking to father was greater among girls than boys in all countries/regions, while difficulty in talking to mother was almost the same for both boys and girls. Difficulty in talking to either parent increased with age but not with overweight (Table 2). Among boys the highest rates in difficulty in talking to father were found in Estonia (43.6%), Belgium (BE-VLG) (41.0%), USA (40.7%), and Czech Republic (40.5%), while among girls the highest rates were found in USA (61.9%), Estonia (60.0%), and Wales (55.8%). Among boys the highest rates in difficulty in talking to mother were found in USA (29.4%), Czech Republic (27.2%), and Wales (23.9%) while among girls the highest rates were found in USA (28.4%), Canada (26.4%) and Estonia (24.4%) (Table 2).

**Table 2:** Communication with father and with mother reported as difficult by gender, age and weight status

**Country**	**Communication with father (difficult)**								**Communication with mother (difficult)**							
	
	**N**	**Boys** **%**	**Girls** **%**	**11 y** **%**	**13 y** **%**	**15 y** **%**	**Non-Over weight** **%**	**Over weight %**	**N**	**Boys** **%**	**Girls** **%**	**11 y** **%**	**13 y** **%**	**15 y** **%**	**Non-Over weight** **%**	**Over weight** **%**
Belgium-Flanders	6151	41.0	52.3***	37.7	46.6***	57.0***	46.3	51.0*	6247	22.8	24.0	17.0	22.6***	31.1***	22.8	25.2
Canada	4239	35.0	55.2***	34.7	49.9***	56.3***	45.7	48.4	4237	21.4	26.4***	17.7	24.3***	32.5***	23.3	26.0
Switzerland	4614	31.8	47.2***	28.6	42.2***	47.2***	39.1	46.5**	4577	19.4	20.3	14.8	19.3***	24.6***	18.7	26.4***
Croatia	4344	28.2	42.8***	22.6	36.2***	48.0***	36.6	32.2	4347	15.2	14.3	9.1	14.7***	20.2***	14.3	14.3
Czech Republic	4744	40.5	55.1***	41.1	49.8***	53.1***	48.6	44.1	4842	27.2**	23.7	22.0	27.1***	27.0***	25.3	26.2
Germany	5428	35.9	55.2***	36.4	47.6***	54.3***	45.8	47.1	5411	19.4	19.9	15.6	18.3*	25.8***	19.4	20.6
Denmark	4510	35.3	51.7***	34.6	46.9***	51.8***	43.7	45.8	4485	22.0	23.6	15.0	25.6***	29.0***	23.2	22.5
Estonia	3978	43.6	60.0***	40.7	53.7***	61.1***	52.4	51.1	3978	23.2	24.4	16.4	23.8***	31.3***	24.0	22.1
Finland	5293	26.5	49.7***	28.6	39.2***	47.2***	38.2	37.2	5194	16.1	19.5**	10.9	19.4***	23.8***	17.5	19.0
France	7819	27.9	42.3***	27.6	34.9***	43.2***	36.0	35.8	7871	16.0	18.3**	12.5	16.7***	22.2***	17.7	15.8
Greece	3708	31.4	56.4***	30.7	46.3***	54.4***	44.2	44.5	3631	23.1**	19.6	13.5	20.6***	29.0***	20.9	22.8
Italy	4337	32.9	52.5***	32.3	46.4***	51.9***	43.6	41.0	4294	20.0	21.1	14.0	21.1***	28.0***	20.9	19.3
Latvia	3185	34.7	55.0***	35.3	48.0***	53.9***	45.9	44.6	3247	21.5	22.5	18.2	22.1*	25.6***	22.0	22.0
Macedonia	4080	16.1	27.4***	15.4	24.2***	25.6***	21.8	18.7	3947	13.9	12.2	13.6	12.8	12.3	12.0	13.3
Netherlands	4208	19.2	31.8***	17.7	27.3***	32.5***	24.9	29.7	4214	8.8	11.7**	6.0	11.2***	14.0***	9.9	11.4
Norway	4910	30.5	45.2***	25.2	37.1***	50.9***	37.6	41.7	4916	17.9	16.4	10.6	14.9***	26.3***	16.8	21.3**
Poland	6231	23.5	32.3***	19.6	25.9***	37.7***	27.9	27.1	6277	10.4	9.0	5.0	9.3***	14.9***	9.6	10.7
Portugal	2862	29.7	52.3***	35.5	44.2***	46.3***	40.6	41.8	2851	19.1	20.4	15.5	22.4***	22.7***	18.9	21.4
Russia	7920	33.2	47.9***	33.5	42.6***	46.9***	41.1	42.3	7956	17.6	18.3	13.1	18.2***	22.5***	17.9	21.7*
Sweden	3850	21.7	39.8***	19.9	31.3***	43.0***	30.8	33.2	3823	12.1	13.9	6.8	13.8***	19.5***	13.1	13.6
Slovenia	3880	14.6	25.6***	14.0	20.5***	27.7***	19.9	21.5	3848	7.4	8.5	4.6	8.7***	11.4***	7.8	8.7
Ukraine	4010	32.7	46.2***	28.3	39.2***	49.2***	40.9	41.3	4022	12.4	11.7	6.9	9.9**	17.7***	12.4	11.1
USA	4921	40.7	61.9***	45.2	51.7***	58.6***	51.9	51.5	4869	29.4	28.4	20.8	29.2***	35.4***	28.4	29.9
Wales	3815	37.4	55.8***	39.7	48.0***	52.1***	46.5	47.4	3799	23.9	22.9	17.9	23.2***	30.0***	22.8	28.3**

References: Age = 11y, Gender = boys, Weight status = non-overweight

Overweight and non-overweight were calculated using international cutoffs for BMI. BMI was calculated based on self reported weight and height

* *p *< 0.05, ** *p *< 0.01, *** *p *< 0.001

### Association between body weight dissatisfaction and communication with parents by gender and country/region

A relationship between body weight dissatisfaction and communication with parents was more common among girls than boys. Among boys, body weight dissatisfaction was positively associated with difficulty in talking to father in 14 out of 24 countries/regions while difficulty in talking to mother was positively associated with body weight dissatisfaction in only two countries (Estonia and Netherlands). Among girls, body weight dissatisfaction was positively associated with difficulty in talking to both father and mother in 19 of the 24 countries examined (Table 3).

**Table 3:** Weight dissatisfaction percentages, odds ratios and confidence intervals by gender and parents' communication

**Country**	**Boys**								**Girls**							
	
	**Communication with father**				**Communication with mother**				**Communication with father**				**Communication with mother**			
	
	**Easy** **%**	**Difficult** **%**	**OR**	**95%CI**	**Easy** **%**	**Difficult** **%**	**OR**	**95%CI**	**Easy** **%**	**Difficult** **%**	**OR **	**95%CI**	**Easy** **%**	**Difficult** **%**	**OR**	**95%CI**
Belgium-Flanders	21.5	27.1	1.26	(1.00–1.59)	23.1	26.0	1.07	(0.82–1.41)	33.4	46.3	1.52***	(1.26–1.83)	36.9	50.1	1.51***	(1.22–1.87)
Canada	25.0	32.1	1.25	(0.92–1.68)	25.6	34.4	1.35	(0.96–1.91)	33.5	50.9	1.76***	(1.39–2.23)	39.6	53.5	1.29*	(1.00–1.67)
Switzerland	20.5	29.2	1.47*	(1.09–1.97)	22.3	27.1	0.92	(0.65–1.31)	36.1	50.0	1.31*	(1.04–1.65)	39.2	57.3	1.89***	(1.43–2.49)
Croatia	32.9	37.4	1.29	(0.98–1.70)	33.5	36.5	1.15	(0.81–1.63)	44.9	59.7	1.57***	(1.24–1.97)	49.2	63.5	1.41*	(1.02–1.95)
Czech Republic	31.5	38.4	1.46**	(1.16–1.84)	33.4	37.7	1.05	(0.81–1.35)	57.5	65.8	1.29*	(1.05–1.58)	60.0	68.2	1.32*	(1.03–1.68)
Germany	28.4	34.6	1.28*	(1.02–1.61)	29.9	34.9	1.24	(0.94–1.63)	36.9	53.2	1.67***	(1.36–2.05)	43.7	56.3	1.44**	(1.13–1.84)
Denmark	26.4	32.0	1.42*	(1.06–1.90)	27.1	33.6	1.34	(0.96–1.85)	38.6	51.9	1.31*	(1.03–1.68)	41.4	58.8	2.12***	(1.59–2.81)
Estonia	16.7	22.0	1.31	(0.99–1.74)	17.7	23.7	1.41*	(1.02–1.94)	32.6	48.2	1.53***	(1.22–1.93)	37.6	55.2	1.68***	(1.31–2.16)
Finland	22.0	24.8	1.11	(0.83–1.49)	22.0	26.4	1.22	(0.86–1.73)	33.0	48.4	1.66***	(1.35–2.04)	37.2	54.4	1.70***	(1.33–2.19)
France	27.3	32.0	1.27*	(1.02–1.58)	28.7	29.1	0.89	(0.67–1.17)	44.7	58.5	1.51***	(1.27–1.79)	47.8	62.3	1.51***	(1.21–1.87)
Greece	33.1	38.3	1.34*	(1.00–1.79)	34.9	35.3	0.99	(0.72–1.37)	46.1	55.7	1.42**	(1.11–1.82)	51.2	54.7	0.85	(0.62–1.15)
Italy	37.1	45.8	1.53**	(1.15–2.03)	39.5	41.9	0.87	(0.62–1.22)	49.3	60.8	1.21	(0.96–1.53)	52.8	64.9	1.53**	(1.16–2.04)
Latvia	12.7	20.0	1.69**	(1.13–2.53)	14.5	18.9	1.29	(0.82–2.01)	32.4	46.8	1.56***	(1.20–2.02)	37.4	49.1	1.41*	(1.05–1.91)
Macedonia	28.6	28.4	1.17	(0.79–1.74)	28.2	30.4	1.36	(0.89–2.07)	46.9	50.8	1.06	(0.81–1.38)	48.3	46.4	0.85	(0.58–1.25)
Netherlands	19.3	26.2	1.59**	(1.12–2.25)	19.8	28.9	1.82*	(1.14–2.91)	29.2	44.1	1.34*	(1.03–1.73)	32.0	50.9	1.56*	(1.09–2.24)
Norway	20.1	29.9	1.59***	(1.23–2.06)	21.9	28.5	1.16	(0.85–1.57)	37.0	56.7	1.77***	(1.44–2.18)	42.9	63.4	1.68***	(1.26–2.24)
Poland	26.9	31.8	1.46**	(1.14–1.87)	27.3	30.8	1.00	(0.71–1.42)	47.2	62.0	1.56***	(1.29–1.90)	50.5	68.1	1.63**	(1.18–2.25)
Portugal	22.1	27.6	1.4	(0.96–2.05)	23.3	27.0	1.06	(0.68–1.65)	39.1	48.3	1.13	(0.85–1.49)	42.1	52.0	1.20	(0.84–1.71)
Russia	13.9	18.7	1.43**	(1.14–1.79)	15.0	17.7	1.11	(0.84–1.47)	31.6	41.1	1.36***	(1.17–1.58)	34.4	43.3	1.24*	(1.03–1.50)
Sweden	18.6	29.0	1.83**	(1.27–2.62)	19.5	28.9	1.25	(0.80–1.94)	30.2	50.8	1.96***	(1.52–2.51)	35.5	55.8	1.59**	(1.13–2.25)
Slovenia	29.6	33.6	1.38	(0.93–2.03)	30.1	31.3	0.71	(0.42–1.20)	52.1	70.7	1.81***	(1.37–2.39)	55.2	71.9	1.44	(0.92–2.25)
Ukraine	14.4	14.2	0.89	(0.63–1.25)	14.1	16.3	1.18	(0.74–1.89)	40.1	48.1	1.08	(0.87–1.35)	42.4	54.4	1.35	(0.97–1.88)
USA	33.6	43.9	1.54***	(1.21–1.96)	35.2	43.3	1.22	(0.94–1.58)	46.2	54.2	1.08	(0.88–1.32)	47.9	60.0	1.59***	(1.28–1.99)
Wales	30.4	38.0	1.19	(0.89–1.57)	31.5	38.7	1.22	(0.89–1.68)	48.3	58.2	1.33*	(1.03–1.71)	50.4	64.8	1.64**	(1.21–2.21)

Binary logistic regression analysis controlling for communication with other parent, age and BMI. Communication with mother reference: easy, Communication with father reference: easy

* *p *< 0.05, ** *p *< 0.01, *** *p *< 0.001

OR: Odds Ratio, CI: Confidence Interval

*Weight dissatisfaction*: either dieting or perceiving a need to diet

## Discussion

Weight problems and dieting have increased among adolescents and are a focus of concern among health professionals worldwide [3]. Previous studies have shown that several demographic characteristics [26], risk behaviors [27,28] and psychosocial factors [3,27,29] are associated with dieting to lose weight. This study expands previous studies in examining the association between body weight dissatisfaction (dieting or perceived a need to diet), and communication with parents (talking to father/mother), in a large sample of adolescents in 24 countries across Europe, Canada, and USA.

### Body weight dissatisfaction

Body weight dissatisfaction was found to be more common among girls, highly prevalent in early adolescence and increased in prevalence with age. These findings are consistent with a previous longitudinal study showing a significant increase in body dissatisfaction among girls during early adolescence [9]. Results from another clinical study demonstrated that across all stages of development, girls were more likely to adopt strategies to lose weight, whereas boys were more likely to adopt strategies to increase muscle [23]. Other research suggests that dieting behavior increases throughout adolescence. Some investigators have found evidence that dieting behaviors at a young age may be a risk factor for eating disorders [30]. Studies indicate that the prevalence of body image concerns and eating problems is high among young adolescent girls [31], and early adolescence has been identified as a vulnerable time for girls to develop disordered eating because of the normative challenges associated with that period of development (e.g. physical changes associated with puberty and increased desire for peer acceptance) [31].

### Difficulty in talking to parents

Our findings indicate that difficulty in talking to father was more prevalent than difficulty in talking to mother, and difficulty in talking to parents was more common among girls than among boys and increased with age. Adolescents prefer sharing information with their mothers rather than fathers; this may be because mothers are more likely to emphasize a conversation orientation over traditional values in communication with their children [32].

A population – based study in US found that one-fourth of girls and boys felt unable to talk to their mother about problems, and over half of girls and one-third of boys did not feel it was easy to talk to their father [19]. These results are consistent with the findings from the current study; about one-half of girls and one-third of the boys in most countries did not feel that they could talk to their father. Our findings are also consistent with previous research indicating that younger boys and girls are more likely than older boys and girls to talk to their mother and ask them about health issues [14]. Mothers also tend to serve as the primary source for health information. In one study, 41.7% of the boys and more than half of the girls (58.4%) identified their mother as the primary resource for health care information [14]. In addition, the nature of parent talk may be different for mothers and fathers; fathers and mothers talk about different topics. Given these differences, it is not surprising that Pipp et al [33] reported that children often felt closer and more attached to mothers than to fathers.

### Association between body weight dissatisfaction and communication with parents'

Results from this study show that adolescents' perceptions of difficulty in talking to parents (especially difficulty in talking to father) were significantly associated with body weight dissatisfaction. These results are consistent with previous studies in which low parental communication and caring were associated with unhealthy weight control [34], body dissatisfaction, depression, and low self-esteem [19]. Also in a prospective study a positive relationship with the mother was significantly associated with increased body satisfaction [1].

Problems in parent-child communications are an indicator of low parental involvement in the adolescents' life and parental involvement has been found to be associated with greater social competence, autonomy, positive attitudes toward school and work, academic achievement, and self-esteem, as well as with less depression, less misconduct at school, and less delinquency and drug use [35-38]. Parental coldness and control have also been associated with poorer mental health [39,40].

A possible mechanism to explain the relationships found might be that warm interaction and responsiveness set the stage for developing compliance and internalized controls while limit-setting and discipline may be less effective in the absence of a positive, warm parent-child relationship [41].

Gender differences in the relationship between communication with parents and body weight dissatisfaction are particularly notable in this study. Among girls, communication with both mother and father were related to body weight dissatisfaction, while among boys, only communication with father was related to dissatisfaction with body weight. Fathers have been reported to be an important influence for boys in terms of both losing weight and increasing muscle mass [23].

As fathers are particularly likely to be involved with sons [42], the positive effects of father-child relationships in middle childhood might be of greater importance for boys. Positive associations between parent-child communication and body dissatisfaction were found in many but not all countries included in the analysis. This reveals that there might be cultural differences in the parent-child relationships and their effects. As in infancy [43], in middle and late childhood there might be cultural differences in the kind of relationships with parents. Children tend to be more or less distressed by particular events depending on experiences and expectations within their culture. "Variations across cultures in the expression of negative emotions and expectations of autonomy and independence may influence both the activation and deactivation of the attachment behavioural system" [44].

Most studies on the effects of parent-child relationship have focused on the mother-child relationships [45]. When fathers' influence has been considered, the emphasis has frequently been on fathers' absence, for example, in families of divorce. The study on the relationship between as well communication with father and mother and the association with body dissatisfaction adds to the recent literature on the positive effect father-child relations can have also in an area often considered as a female topic.

Parent-child communication is one aspect of positive parenting. Our data did not permit to explore if the communication within the family had an independent effect of e.g. parental control or if involvement can mediate the effects of other aspects of parenting as monitoring or limit setting. Also the question if the quantity of parent-child contact is more important than the quality of the contact still needs further research.

### Limitations

The survey items assessing parent – adolescent communication were limited in scope as part of a large survey on health risk behaviours and thus cannot provide an in-depth exploration of the parent – adolescent relationship. The study also relied exclusively on a single informant, the adolescent, and could have been enriched with corroborating information from parents and siblings. The cross-sectional design of the study precluded any conclusions about the direction of causality between variables. Finally, data on weight and height were self-reported which can produce lower prevalence estimates of overweight and obesity [46].

## Conclusion

Difficulty in talking to mother was associated with body weight dissatisfaction among girls but not among boys, while difficulty in talking to father was associated with body weight dissatisfaction for both boys and girls. These findings suggest that parental involvement might be an important aspect in the adolescents' physical and emotional development and in the prevention of body weight dissatisfaction. More longitudinal, multiple perspective (parent and child), and interventional studies are needed. Professionals working with adolescents and their families should help adolescents to have a healthy weight and a positive body image and promote effective parent – adolescent communication.

## Competing interests

The authors declare that they have no competing interests.

## Authors' contributions

HA: drafted the manuscript, developed its design, and performed the statistical analyses. CV: the study design and helped to draft the manuscript. FE: revised the manuscript, proof reading and critical comments. ZA: revised the manuscript critically. LM: participated in the design of the study and revised the manuscript critically. TN: revised the manuscript critically and edited the final version. NA: revised the manuscript, proof reading and critical comments. KA: constructive commentes. KO: constructive commentes. All authors have read and approved the final manuscript.

## Pre-publication history

The pre-publication history for this paper can be accessed here:



## References

[B1] Barker ET, Galambos NL (2003). Body dissatisfaction of adolescent girls and boys: Risk and resource factors. Journal of Early Adolescence.

[B2] Smolak L, Levine M, Striegel-Moore R (1996). The Developmental Psychology Of Eating Disorders.

[B3] French SA, Jeffery RW (1994). Consequences of Dieting to Lose Weight – Effects on Physical and Mental-Health. Health Psychology.

[B4] Ojala K, Vereecken C, Välimaa R, Currie C, Villberg J, Tynjälä J (2007). Attempts to lose weight among overweight and non-overweight adolescents: a cross-national survey. Int J Behav Nutr Phys Act.

[B5] Strauss RS (1999). Self-reported weight status and dieting in a cross-sectional sample of young adolescents – National Health and Nutrition Examination Survey III. Archives of Pediatrics & Adolescent Medicine.

[B6] Altabe M (1998). Ethnicity and body image: Quantitative and qualitative analysis. International Journal of Eating Disorders.

[B7] Siegel JM, Yancey AK, Aneshensel CS, Schuler R (1999). Body image, perceived pubertal timing, and adolescent mental health. Journal of Adolescent Health.

[B8] Presnell K, Bearman SK, Slice E (2004). Risk factors for body dissatisfaction in adolescent boys and girls: A prospective study. International Journal of Eating Disorders.

[B9] Bearman SK, Presnell K, Martinez E, Stice E (2006). The skinny on body dissatisfaction: A longitudinal study of adolescent girls and boys. Journal of Youth and Adolescence.

[B10] Collins ME (1991). Body Figure Perceptions and Preferences Among Preadolescent Children. International Journal of Eating Disorders.

[B11] Mccabe MP, Ricciardelli LA (2001). Parent, peer, and media influences on body image and strategies to both increase and decrease body size among adolescent boys and girls. Adolescence.

[B12] Neumark-Sztainer D (2005). Preventing the broad spectrum of weight-related problems: Working with parents to help teens achieve a healthy weight and a positive body image. Journal of Nutrition Education and Behavior.

[B13] Gittleman MG, Klein MH, Smider NA, Essex MJ (1998). Recollections of parental behaviour, adult attachment and mental health: mediating and moderating effects. Psychological Medicine.

[B14] Ackard DM, Neumark-Sztainer D (2001). Health care information sources for adolescents: Age and gender differences on use, concerns, and needs. Journal of Adolescent Health.

[B15] Moreno A, Thelen MH (1993). Parental Factors Related to Bulimia-Nervosa. Addictive Behaviors.

[B16] Berndt J, Hestenes S, Smolak L, Levine M, Striegel-Moore R (1996). The Developmental Course of Social Support: Family and Peers. The Developmental Psychopathology Of Eating Disorders: Implications for Research, Prevention, and Treatment.

[B17] Ewell F, Smith S, Karmel M, Hart D, Smolak L, Levine M, Striegel-Moore R (1996). The Sense of Self and Its Development: A Framework for Understanding Eating Disorders. The Developmental Psychopathology Of Eating Disorders: Implications for Research, Prevention, and Treatment.

[B18] Currie C, Hurrelmann K, Settertobulte W, Smith R, Todd J, (eds) (2000). Health and health behaviour among young people. WHO Policy Series: international report Health policy for children and adolescents Issue 1, WHO Regional Office for Europe. Copenhagen.

[B19] Ackard DM, Neumark-Sztainer D, Story M, Perry C (2006). Parent-child connectedness and behavioral and emotional health among adolescents. American Journal of Preventive Medicine.

[B20] Fulkerson JA, Neumark-Sztainer D, Story M (2006). Adolescent and parent views of family meals. Journal of the American Dietetic Association.

[B21] Boutelle KN, Fulkerson JA, Neumark-Sztainer D, Story M, French SA (2007). Fast food for family meals: relationships with parent and adolescent food intake, home food availability and weight status. Public Health Nutrition.

[B22] Neumark-Sztainer D, Eisenberg M, Fulkerson JA, Story M, Larson N (2008). Family Meals and Disordered Eating in Adolescents. Arch Pediatr Adolesc Med.

[B23] Mccabe MP, Ricciardelli LA (2005). A prospective study of pressures from parents, peers, and the media on extreme weight change behaviors among adolescent boys and girls. Behaviour Research and Therapy.

[B24] Currie C, Samdal O, Boyce W (2002). Health behaviour in school-Aged Children: a WHO Cross-National Study. Research protocol for the 2001–2002 survey Edinburgh, Scotland.

[B25] Cole TJ, Bellizzi MC, Flegal KM, Dietz WH (2000). Establishing a standard definition for child overweight and obesity worldwide: international survey. British Medical Journal.

[B26] Neumark-Sztainer D, Story M, Falkner NH, Beuhring T, Resnick MD (1999). Sociodemographic and personal characteristics of adolescents engaged in weight loss and weight/muscle gain behaviors: Who is doing what?. Preventive Medicine.

[B27] Crow S, Eisenberg ME, Story M, Neumark-Sztainer D (2006). Psychosocial and behavioral correlates of dieting among overweight and non-overweight adolescents. Journal of Adolescent Health.

[B28] Mellin AE, Neumark-Sztainer D, Story M, Ireland M, Resnick MD (2002). Unhealthy behaviors and psychosocial difficulties among overweight adolescents: The potential impact of familial factors. Journal of Adolescent Health.

[B29] French SA, Story M, Downes B, Resnick MD, Blum RW (1995). Frequent Dieting Among Adolescents – Psychosocial and Health Behavior Correlates. American Journal of Public Health.

[B30] Gralen SJ, Levine MP, Smolak L, Murnen SK (1990). Dieting and Disordered Eating During Early and Middle Adolescence – do the Influences Remain the Same. International Journal of Eating Disorders.

[B31] Mcvey GL, Pepler D, Davis R, Flett GL, Abdolell M (2002). Risk and protective factors associated with disordered eating during early adolescence. Journal of Early Adolescence.

[B32] Miller-Day MA (2002). Parent-adolescent communication about alcohol, tobacco, and other drug use. Journal of Adolescent Research.

[B33] Pipp S, Jennings S, Shaver P, Lamborn S, Fischer KW (1985). Adolescents Theories About the Development of Their Relationships with Parents. Journal of Personality and Social Psychology.

[B34] Neumark-Sztainer D, Wall MM, Story M, Perry CL (2003). Correlates of unhealthy weight-control behaviors among adolescents: Implications for prevention programs. Health Psychology.

[B35] Allen JP, Hauser ST (1996). Autonomy and relatedness in adolescent – family interactions as predictors of young adults' states of mind regarding attachment. Development and Psychopathology.

[B36] Parish TS, McCluskey JJ (1992). The relationship between parenting styles and young adults' self-concepts and evaluations of parents. Adolescence.

[B37] Steinberg L, Lamborn SD, Dornbusch SM, Darling N (1992). Impact of parenting practices on adolescent achievement: Authoritative parenting, school involvement, and encouragement to succeed. Child Development.

[B38] Doyle BA, Moretti MM, Brendgen M, Bukowski W Parent-child relationships and adjustment in adolescence. Findings from the HBSC Cycle 3 and NLSCY Cycle 2 studies.

[B39] Collins NL, Read SJ (1990). Adult attachment, working models, and relationship quality in dating couples. Journal of Personality and Social Psychology.

[B40] Rodgers B (1996). Reported parental behaviour and adult affective symptoms. 1. Associations and moderating factors. Psychological Medicine.

[B41] Maccoby EE, Martin JA, Hetherington (1983). Socialization in the context of the family: Parent-child interaction. Socialization, personality, and social development.

[B42] Pleck JH, Lamb (1997). Paternal involvement: Levels, sources, and consequences. The role of the father in child development.

[B43] van IJzendoorn MH, Sagi A, Cassidy J, Shaver PR (1999). Cross-cultural patterns of attachment: Universal and contextual dimensions. Handbook of attachment: Theory, research, and clinical applications.

[B44] Dwyer KM (2005). The meaning and measurement of attachment in middle and late childhood. Human Development.

[B45] Lamb ME (1997). The role of the father in child development.

[B46] Elgar FJ, Roberts C, Tudor-Smith C, Moore L (2005). Validity of self-reported height and weight and predictors of bias in adolescents. Journal of Adolescent Health.

